# Internal marketing analysis for improving the internal consumer satisfaction and customer orientation of employees in private-owned sports center

**DOI:** 10.1371/journal.pone.0286021

**Published:** 2023-08-10

**Authors:** Shi Qi Xu, Lian Zhou, Seong Hun Kim, Dong-Hwa Chung, Zhen Li

**Affiliations:** 1 Department of Sports Convergence Technology, Sangmyung University, Seoul, South Korea; 2 Department of Physical Education, Chung-ang University, Seoul, South Korea; SGH Warsaw School of Economics: Szkola Glowna Handlowa w Warszawie, POLAND

## Abstract

With the concept of “healthy lifestyle” deeply rooted in people’s minds, the sports service industry is thriving, which has resulted in intense competition. The sports service industry must improve its service quality to be competitive. Customer orientation is the key factor for enterprises to gain competitive advantage. With the in-depth understanding of internal marketing in the service industry. Managers have realized that treating employees as internal consumer is a good way to improve their satisfaction and gain customer orientation. However, what internal marketing strategies will have a positive effect on internal consumer satisfaction and customer orientation of private-owned sports center employees are still unclear. In this investigation, a total of 326 employees from the private-owned sports center were used to investigate the effects of internal marketing strategies on internal consumer satisfaction and customer orientation. All employees were asked to complete a questionnaire on 5-point scale. A path model was used to investigate the direct and indirect effects of hypothetical measurements on internal consumer satisfaction and customer orientation. The findings suggested that internal communication, administrative support, and educational training were important factors affecting internal consumer satisfaction and customer orientation. We concluded that the implementation of internal marketing strategies could improve internal consumer satisfaction and customer orientation, and higher levels of internal consumer satisfaction will encourage employees to have higher degrees of customer orientation. Therefore, the implementation of internal marketing strategy was beneficial to the development of private-owned sports centers.

## Introduction

The private-owned sports center is an infrastructure that provides convenient and diversified exercise programs at a reasonable price. With the concept of “healthy lifestyle” deeply rooted in people’s minds, the development of private-owned sports centers is thriving [1]. Simultaneously, the spread of sports culture promoted by government support also boosts the development of private-owned sports centers.

However, from the perspective of market supply, the excessive supply of private-owned sports centers makes the managers of sports centers encounter an intensely competitive market environment [2]. How to improve the benefits of enterprises in this environment has become a key issue for managers.

The benefits of enterprises are affected by internal and external factors. In the case that external factors are not easy to change, more and more managers are starting to pay attention to the management of internal factors [3, 4]. In the service process of sports center, employee, also known as internal consumer, has a close relationship with customers [5]. The quality of service provided by employees plays a key role in affecting customers’ consumption mood. Higher service quality will make customers have higher consumption intention. Therefore, better performance will be achieved by understanding and satisfying individual needs to motivate internal customers [6]. Additionally, treating employees as initial customers and instilling service consciousness or customer first concept in them can optimize their service attitude. The strategy of satisfying employees’ needs and customer-centered service objectives is called internal marketing strategy [7, 8]. From the perspective of marketing, the implementation of internal marketing strategy helps employees to have a positive attitude and is beneficial to the development of enterprises [7, 8]. Therefore, the establishment of managing means centered on internal marketing management is necessary to make private-owned sports centers gain a stronger competitive advantage.

The successful implementation of internal marketing strategy can improve service quality by improving the satisfaction of internal consumers, so as to achieve the purpose of improving customer satisfaction [9, 10]. So-called internal consumer satisfaction can be defined as the cheerful and positive emotional state of employees when they are engaged in work-related activities [10]. Internal consumer satisfaction plays a decisive role in commercial activities. It is reported that the implementation of internal marketing strategy can improve employees’ satisfaction and loyalty, and promote customers’ repeated purchase behavior [4, 11–13].

Moreover, the intimate relationship between employees and customers is established by meeting the demands of customers. Therefore, the behavior of internal consumers has the characteristics of customer orientation [14]. That is, in the process of contacting customers, employees strive to understand the demand of customers and try their best to meet their demands [15, 16]. Therefore, customer orientation plays an important role in enterprise development in a competitive environment [17].

By implementing internal marketing strategies, the sports center can improve internal consumer satisfaction and customer orientation, thus improving customer satisfaction and realizing business behavior, which is of great significance to maintaining high competitiveness [14].

However, studies related to the development of private-owned sports centers are mainly focused on the aspects of customer satisfaction evaluation [18], customer loyalty [19], repurchase intention of customers [20], and the investigation of customer situation [21]. Currently, studies on the effects of internal marketing strategy on the marketing behavior of private-owned sports centers are still limited. Future development of private-owned sports centers will benefit from the implementation of internal marketing strategies. Therefore, the objective of this study was to investigate the effects of implementing internal marketing strategy in private-owned sports centers on internal consumer satisfaction and customer orientation, which contributed to providing a new strategy for the development of private-owned sports centers.

### Literature review and research hypothesis

Fierce market competition makes the marketing strategy of service industries change from passive to active. At present, many enterprise marketing researches focus on the relationship between enterprise activities and external customers [22, 23]. Limited studies have investigated the role of employees in corporate marketing activities [24]. This is unfortunate, because employees are not only the main stakeholder group of the enterprise, but also play a frontline and interactive role in affecting the service experience of customers [25]. Employees as a crucial factor in maintaining company’s value have not been given enough attention. Enterprise managers have gradually realized that paying attention to the needs of employees is very important to improve their job satisfaction, so as to better connect enterprises with external customers. Chiu et al. [26] suggested that internal marketing was an effective measure to improve the job satisfaction of employees in public sports center. Huang & Chen [27] reported that internal marketing was associated with the customer orientation of full- and part-time service employees at public sports centers. Chiu et al. [28] investigated the relationship between internal marketing and work performance in municipal sports centers, they suggested that internal marketing had positive effects on work performance. Therefore, internal marketing is an effective strategy to improve work satisfaction, customer orientation, and work performance. Under the framework of service industries, internal marketing is defined as a strategy to regard employees as internal consumers and actively respond to their needs [29]. In this study, the role of the sports center’s employee is defined as internal consumer. However, the above studies are examined in the context of public sports centers, which is different from this study. As the private-own sports centers are more profit-based, the role of internal marketing can be more important to improve internal consumer satisfaction and customer orientation.

### Internal marketing

Since 1990s, concepts of marketing have been gradually applied to human resource management, and the concept of internal marketing came into being [30, 31]. From the perspective of marketing, internal marketing is a strategic activity that integrates marketing with human resource management, aiming at helping employees to have a positive attitude, so as to realize the sustainable competitive advantage for enterprises [7, 8, 32]. Therefore, this strategy requires organizations to regard employees as internal consumers and constantly meet the needs of employees [33]. In service organizations, employees are at the forefront of communication activities with customers [34, 35]. According to marketing theories, internal marketing emphasizes the importance of employees’ satisfaction and regards their work as internal products [36]. The goal of internal marketing is to develop and motivate employees. The function of internal marketing is based on the supposition that motivated employees comply with organizational policies and decision-making. This benefits the organization as employees understand organizational goals, empowering employees to act, and fulfill their roles. Therefore, these dedicated employees can promote the development of the organization [37]. Internal marketing recognizes the value of employees [38], which is conducive to increasing employees’ enthusiasm and then helps to enhance their human and social capital. The company will develop better with the motivation of employees increased. Many studies suggest that internal marketing activities are the primary tools to ensure employee retention, customer satisfaction, and profitability [39–42]. It has been stated that frontline employees working in the field of sports and fitness usually lack motivation and interaction with organizations and customers due to limited training and the minimum wage culture [43, 44]. Therefore, the implementation of internal marketing is expected to help employees cultivate a sense of ownership for their work [44]. Internal marketing activities can provide a supportive environment for employees, help employees build a sense of responsibility and belonging to their work, and their level of identification with an organization, thus improving their performance [43, 45, 46]. The importance of internal marketing in the development of sports industry has been fully demonstrated [26, 27, 47–49].

### Internal consumer satisfaction

Emotional state is an important factor affecting employees’ behavioral intentions [50]. Their emotional state is closely related to their work satisfaction [10]. High satisfaction of employees with their work will produce positive emotions. From the perspective of marketing, all employees in an enterprise are internal consumers who serve external customers. Therefore, employees’ satisfaction with work is essential for the satisfaction of external customers [29]. It has to be recognized, however, that the sports service industry is distinct from other service industries. Consumers at sports centers frequently interact with service staff since they completely participate in tasks during the service process [51]. Therefore, good internal consumer satisfaction ensures that they have a good emotional state, which is conducive to improving the quality of service.

### Customer orientation

Customer orientation refers to the predisposition of employees to meet customers’ needs in their work environment [52]. This predisposition is very important, because customer orientation is the basic feature of quality service [53]. In the service industry, employees with high customer orientation play an important role in affecting the company’s profitability and realizing the company’s competitive advantage [54, 55]. In the process of contacting consumers, employees with high customer orientation will strive to understand the needs of consumers and try their best to meet their needs [15, 16], which is conducive to establishing long-term relationships between potential customers and enterprises [56]. As stated by Donavan et al. [57], highly customer-oriented employees are more suitable for service jobs. Therefore, in the increasingly competitive service-oriented market, it is crucial to create a customer-oriented business culture for the successful operation of enterprises [58]. The goal of sports clubs is to promote and cultivate interest in a particular sport or physical activity. During the exercise, customers have many demands, such as finding ways to use equipment, understanding exercise precautions, etc. Internal consumers should solve these demands in time. As mentioned by Ng [59], sports clubs should set customer-focused actions and beliefs for achieving long-term development.

### Internal consumer satisfaction & customer orientation

Internal consumer satisfaction is associated with customer-oriented behavior [57, 60] and customer-friendly behavior [61]. High levels of work satisfaction indicate that employees have higher degrees of customer orientation [10, 60, 62], thus providing better services to customers and improving customer satisfaction [63]. Therefore, we hypothesized that internal consumer satisfaction of employees in private-owned sports centers had positive effects on customer orientation (Hypothesis 1).

If hypothesis 1 was supported, the satisfaction of internal consumers was significantly related to customer orientation. Therefore, the antecedents of internal consumer satisfaction would indirectly affect customer orientation. Proceeding from this logic, all the hypotheses put forward in part (b) below were corollaries to part (a).

As a strategic management strategy, internal marketing is an effective means to improve internal consumer satisfaction and customer orientation [64]. According to Woodworth’s [65] stimulation organism-reaction model, employees are organisms with expectations and motivations. After receiving stimulation, individuals first transform it through expectations and motivations, and eventually chooses a response. That is, when internal marketing fails to bring a high level of internal consumer satisfaction, internal consumer will not respond with high customer orientation. Typical internal marketing activities can be identified and classified to provide a framework for evaluating their implications and effectiveness [66]. In the present study, the internal marketing strategies are divided into five areas, reflecting the categories conceptualized by Gronroos [66], i.e. internal communication, administrative support, encouraging system, educational training, and authority appointment. It is important to check each of these measurements to examine their contribution to internal consumer satisfaction and their corresponding effect on customer orientation.

### Internal communication

Two-way communication between managers and employees not only strengthens the support of management, but also provides feedback for employees to improve their work performance [66]. Employees need to understand the needs of customers, their organization, and the importance of their contribution to the organization and customers. Effective communication within an enterprise is a necessary condition for developing internal consumer satisfaction and customer orientation [67, 68]. In an investigation of sports clubs, Roșca [69] suggested that good communication between club managers and athletes had a great influence on athletes’ performance, and thus enhanced the satisfaction of athletes and their fans. In this environment, athletes’ performances are provided for fans, and the role of athletes is a server. Effective communication seems to be an effective measure to create the best working environment. If communication is clear and focused on the mission, it can promote participation, increase the trust of managers and colleagues, and enhance loyalty [69]. Therefore, we hypothesized that internal communication positively affected internal consumer satisfaction (Hypothesis 2a) and customer orientation (Hypothesis 2b).

### Administrative support

Managers have the responsibility to create an atmosphere conducive to improving internal consumer satisfaction and developing customer orientation [66]. Managers need to show administrative support by paying attention to employees [70] and responding to their suggestions [71, 72]. The role of administrative support has become a necessary prerequisite for cultivating internal consumer satisfaction and customer orientation [41]. To achieve the success of the sports industry, sound administrative support is necessary. Therefore, we hypothesized that administrative support positively affected internal consumer satisfaction (Hypothesis 3a) and customer orientation (Hypothesis 3b).

### Encouraging system

Encouraging system can motivate employees to adopt new behaviors and attitudes that are beneficial to the development of enterprises. Encourage efforts aimed at providing customers with the best possible service will help to improve their work enthusiasm and service quality [14, 70, 73]. Meyer & Allen [74] stated that employees tend to develop strong commitments when the incentives provided exceed employees’ expectations. Encouraging system has become an important strategy to improve internal consumer satisfaction [10, 75] and customer orientation [67, 76]. Therefore, we hypothesized that encouraging system positively affected internal consumer satisfaction (Hypothesis 4a) and customer orientation (Hypothesis 4b).

### Educational training

For the service industry, employees gaining an understanding of their role and what they should do within a customer-oriented enterprise is important. Educational training can provide employees with professional skills and sensitivity to customer needs to realize customer orientation [77]. Educational training can help employees understand each other’s role relative to others and various functions in the enterprise, thus forming an overall view of service strategy [66]. Additionally, training and educational activities can improve employees’ skills and abilities related to their tasks and development, and enhance their positive attitude and commitment to work [28]. Chiu et al. [28] investigated the relationship between educational training and job performance in a public sports center, they suggested that educational training is an effective measure to improve job performance. Customers evaluate the quality of service according to their interaction with service employees and their behaviors. Consequently, employee knowledge and interpersonal skills are important to the success of a service enterprises [78]. Training and education of employees can be regarded as an investment in employees. Extending the theory of social exchange to service settings suggests that when employers invest more in employees, employees are more likely to reciprocate the benefits they have obtained [79, 80]. Educational training is important for the development of internal consumer satisfaction [9, 75] and customer orientation [76, 81] within an enterprise. Therefore, we hypothesized that educational training positively affected internal consumer satisfaction (Hypothesis 5a) and customer orientation (Hypothesis 5b).

### Authority appointment

Personnel management represents the design and implementation of human resource policies. Customer-oriented employees are an asset. It is necessary to keep these employees by maintaining satisfactory human resource policies [66]. It is more effective to select employees with high customer orientation than to train an employee with these characteristics [66, 77]. Designing and implementing human resource policies, such as effective election practice, can improve internal consumer satisfaction and customer orientation [67, 77]. Therefore, we hypothesized that authority appointment positively affected internal consumer satisfaction (Hypothesis 6a) and customer orientation (Hypothesis 6b).

### Conceptual framework

**Fig 1:**
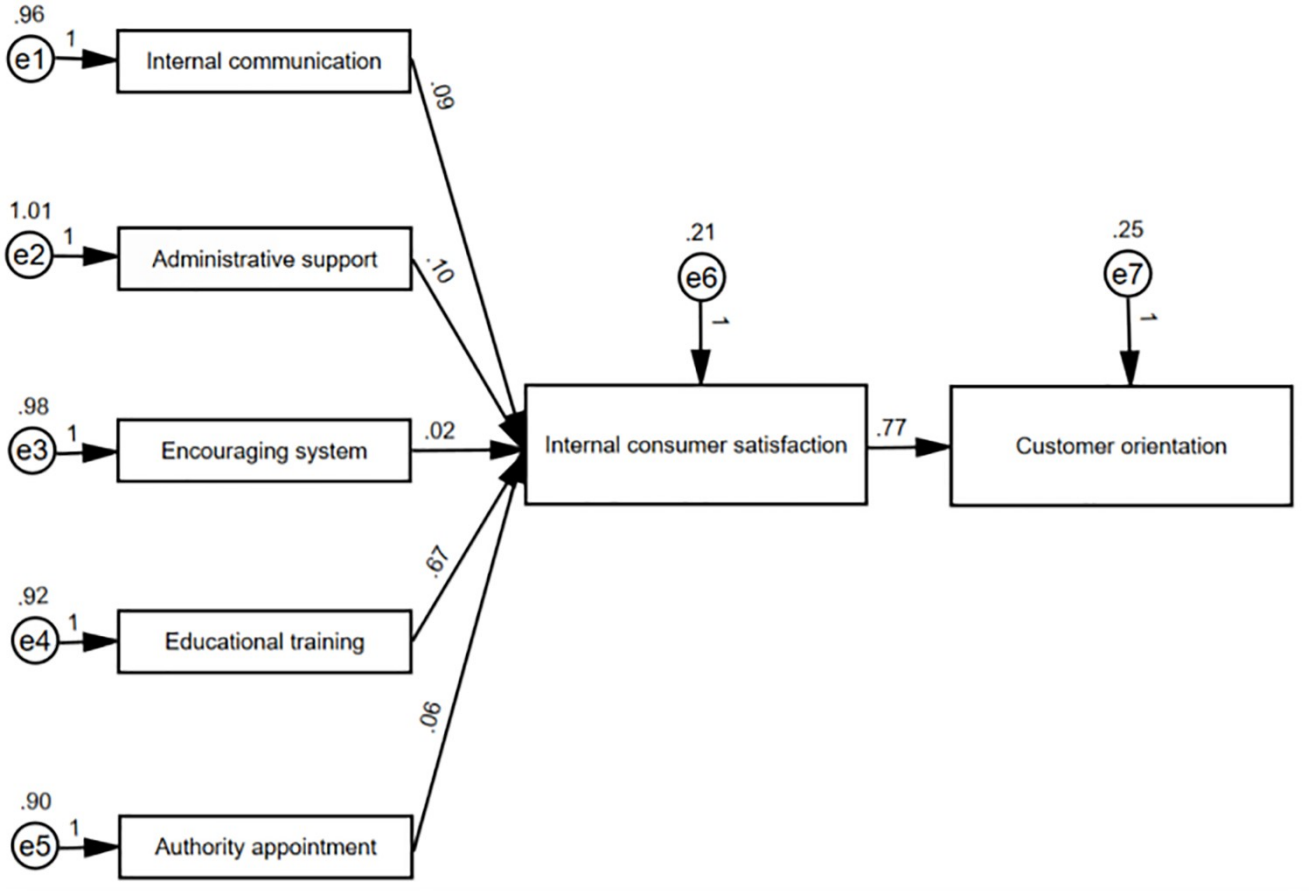
Path analysis (calculated using AMOS). Fitting degree of model: Cmin/df, 1.368; GFI, 0.998; Adjusted GFI, 0.967; Root mean standard error of approximation, 0.034; Tucker-Lewis index, 0.994; Normed fit index, 0.998; PNFI, 0.095.

The purpose of this study is to explore various internal marketing practices through researching employee satisfaction and testing the relationship between internal consumer satisfaction and customer orientation. Based on these assumptions, this study developed a research model, which is shown in Fig 1.

## Materials and methods

The procedure and protocol used in this study have been approved by the ethics committee of Sangmyung University (Seoul, South Korea).

### Survey design and questionnaire

The questionnaire used in this study was administrated and web-programmed by Wen Juan Xing (Changsha Ranxing IT Ltd.) and was spread using WeChat. The questionnaire was structured with closed-ended questions and divided into four parts (S1 Table). Among them, the design of items from section 2 were referred to the studies of Sun [82] and Lee [83]; items from section 3 were referred to the studies of Kim [84] and Lee [19]; items from section 4 were referred to the studies of Lee [19] and Lee [11].

In section 1, we collected the socio-demographic information of the respondents, including age, gender, work experience, and employee type.

Section 2 consists of five measurements, including internal communication, administrative support, encouraging system, educational training, and authority appointment. In the measurement of internal communication, each respondent’s evaluation of the freedom to express one’s wishes, communicate work or other things, understand work-related guidelines and information on work performance results, and actively express one’s intention to the boss were measured on a 5-point scale. In the measurement of administrative support, each respondent’s evaluation of the management in creating an approachable atmosphere, encouraging open communication, allowing employees to participate in the planning and decision-making process, and striving to promote the exchange of information between each other were measured on a 5-point scale. In the measurement of encouraging system, each respondent’s evaluation of company’s reward based on customer evaluation, establishing close customer relationships, and putting forward ideas that will help improve business were measured on a 5-point scale. In the measurement of educational training, each respondent’s evaluation of the frequency of service and job-related education and training, the opportunities to provide continuing education and training, the help of education and training to understand the customers’ present and future requirements, and the value of company’s formal education and training were measured on a 5-point scale. In the measurement of authority appointment, each respondent’s evaluation of the company in allowing employees to have autonomy in job-related decisions, encouraging employees to use their own judgment when solving problems, encouraging innovation, and giving employees a lot of initiative were measured on a 5-point scale.

In section 3, ten items were given on a 5-point scale to understand respondent’s satisfaction with the internal service quality of the company, their work in the company, matching degree between work and competence level, workload, freedom to express opinions on their work, freedom to communicate with their superiors about their work, the treatment level provided by the company, colleagues, and current working environment, as well as overall satisfaction with the company’s work.

In section 4, 14 items were given on a 5-point scale to understand respondent’s evaluation of “customers first”, explain the problem to the customer in detail, take customers’ problems seriously, know what customers need in advance, understand customers’ needs, be good at listening to customers, be interested in customer behavior, recognize and respond to customers’ needs in advance, provide customers with the services they want accurately, provide a good service that customers can trust, provide service at the time appointed with customers, pay individual attention to customers, remember customer’s name, and strive to maintain a good relationship with customers.

### Data collection and analysis

The questionnaire was distributed to the frontline employees of three relatively large-scale private-owned sports centers located on Wuhan, China (120 for Kongzhong sport center; 120 for Cutting Up sport center; 110 for Any sport center). Each of these sports centers has more than 15 branches in Wuhan. The survey was conducted using a convenient sampling method [85]. During the survey period, we visited the above sports centers and invited the respondents face-to-face. A total of 339 responses were received, of which 13 were unusable. A total of 326 questionnaires were then used for the analysis. Personal characteristics of employees were shown in Table 1. All participants were informed of the purpose of this study, and each submitted written informed consent before participating in this study.

**Table 1:** Demographic characteristics of respondents in private-owned sports center.

Respondents	Number	Percentage, %
Total number of volunteers	325	
Gender		
Male	248	76.07
Female	78	23.93
Age		
20–29 years old	227	69.63
30–39 years old	49	15.03
40–49 years old	50	15.34
Work experience		
Less than 1 year	121	37.11
1–2 years	66	20.25
2–3 years	40	12.27
More than 3 years	99	30.37
Employee type		
Formal employee	171	52.45
Informal employee	155	47.55

### Statistical analysis

All data obtained in this study were analyzed by the software of SPSS (version 26.0). Method of frequency analysis was used to analyze the general characteristic of respondents. The validity and reliability of questionnaire were examined via confirmatory factor analysis and Cronbach’s alpha, respectively. The research hypotheses in the proposed model were tested by using the method of structural equation modeling by AMOS. The two-stage test program proposed by Anderson & Gerbing [86] was adopted to estimate measurement and structural models. In the first stage, the items in the research model were estimated by confirmatory factor analysis. In the second stage, structural relationships among the constructs in the research model were estimated to evaluate the structural model and test the research hypotheses. Results were considered significant at *P* < 0.05.

## Results

### Demographic characteristics of respondents

A total of 325 respondents were used in this investigation, of which 76.07% of respondents were male and 23.93% were female. More than half of respondents were younger than 29 years old (69.63%), others were older than 30 years old, of which older than 30 years old but younger than 39 years old were 15.03%, while older than 40 years old but younger than 49 years old were 15.34%. Most of respondents were engaged in private-owned sports center for less than 1 year (37.11%), moreover, working experience for more than 1 year but less than 2 years were 20.25%, more than 2 years but less than 3 years were 12.27%, others were experienced for more than 3 years (30.37%). For employee type, more than half of respondents were formal employee (52.45%) and less than half of respondents were informal employee (47.55%) (Table 1).

### Results of factor analysis

Based on the results of factor analysis of internal marketing (S2 Table), internal consumer satisfaction (S3 Table), and customer orientation (S4 Table), no questions involving low factor loading value were observed. Factors with eigenvalues higher than 1.0 were thus extracted. The factor analysis among these three analyses was reasonable based on the coefficient of Cronbach’s α of all factors. Additionally, according to the results of convergent and discriminant validity statistics, all average variance extracted and composite reliability values for the multi-item scales were greater than 0.6 and 0.8, respectively, indicating a sufficient level of convergent validity for the measurement model.

### Model parameters

Results of the research model were shown in Fig 1. Parameters in model confirmed that the proposed measurement model fitted the data well, of which Cmin/df = 1.368; GFI = 0.998; adjusted GFI = 0.967; root mean standard error of approximation = 0.034; Tucker-Lewis index = 0.994; Normed fit index = 0.998; PNFI = 0.095.

### Indirect and total effects

Total effects were the sum of direct and indirect effects. The concept of total effects was important as it seeks to interpret all changes, including mediating effects, from an independent measurement to a dependent measurement. Therefore, this study can be said to interpret the total effects of each dependent measurement. As shown in Table 2, internal consumer satisfaction was the most powerful antecedent for predicting customer orientation, with the largest total impact (0.864), followed by encouraging system (0.576), internal communication (0.096), and administrative support (0.081). In assessing internal consumer satisfaction, educational training (0.667) was the most significant factor, followed by internal communication (0.111) and administrative support (0.094).

**Table 2:** Results of structural equation modeling.

	Direct effect		Indirect effect		Total effect	
	ICS	CO	ICS	CO	ICS	CO
IC	0.111***	-	-	0.096***	0.111***	0.096***
AS	0.094**	-	-	0.081**	0.094**	0.081**
ES	0.017	-	-	0.015	0.017	0.015
ET	0.667***	-	-	0.576***	0.667***	0.576***
AA	0.065	-	-	0.056	0.065	0.056
ICS	-	0.864***	-	-	-	0.864***
CO	-	-	-	-	-	-

Abbreviation: IC, internal communication; AS, administrative support; ES, encouraging system; ET, educational training; AA, authority appointment; ICS, internal consumer satisfaction; CO, customer orientation.

**P < 0.01;

***P < 0.001

### Hypothesis testing

The results of hypothesis testing indicated that internal communication, administrative support, and educational training had a significant direct effect on internal consumer satisfaction (internal communication → internal consumer satisfaction = 0.111, *P* < 0.001; administrative support → internal consumer satisfaction = 0.094, *P* < 0.01; educational training → internal consumer satisfaction = 0.667, *P* < 0.001) and a significant indirect effect on customer orientation (internal communication → customer orientation = 0.096, *P* < 0.001; administrative support → customer orientation = 0.081, *P* < 0.01; educational training → customer orientation = 0.576, *P* < 0.001). The findings supported hypotheses 2a, 2b, 3a, 3b, 5a, and 5b. However, encouraging system and authority appointment were not statistically significant in predicting internal consumer satisfaction and customer orientation, leading us to reject hypotheses 4a, 4b, 6a, and 6b. Additionally, internal consumer satisfaction was a significant determinant for explaining customer orientation (internal consumer satisfaction → customer orientation = 0.864, *P* < 0.001), supporting hypothesis 1 (Table 3).

**Table 3:** Summary of support for hypothesis.

Hypothesis		t value	Results
Hypothesis 1	Internal consumer satisfaction → Customer orientation	0.864***	Supported
Hypothesis 2a	Internal communication →Internal consumer satisfaction	0.111***	Supported
Hypothesis 2b	Internal communication → Customer orientation	0.096***	Supported^1^
Hypothesis 3a	Administrative support → Internal consumer satisfaction	0.094**	Supported
Hypothesis 3b	Administrative support → Customer orientation	0.081**	Supported^1^
Hypothesis 4a	Encouraging system → Internal consumer satisfaction	0.017	Not supported
Hypothesis 4b	Encouraging system → Customer orientation	0.015	Not supported
Hypothesis 5a	Educational training → Internal consumer satisfaction	0.667***	Supported
Hypothesis 5b	Educational training → Customer orientation	0.576***	Supported^1^
Hypothesis 6a	Authority appointment → Internal consumer satisfaction	0.065	Not supported
Hypothesis 6b	Authority appointment → Customer orientation	0.056	Not supported

^1^result includes indirect effects

**P < 0.01;

***P < 0.001

## Discussion

Results obtained in this study indicated that employees with high work satisfaction have higher customer orientation. Similarly, a study investigated the relationship between internal consumer satisfaction and customer orientation for employees in the National Opera House and Ballet Theatre, and suggested that employees’ customer orientation was positively affected by their work satisfaction [62]. Yoo & Park [87] reported that enhancing hospital employees’ internal consumer satisfaction would increase their customer orientation. Additionally, Lee et al. [88] noted that internal consumer satisfaction was positively related to customer orientation. Therefore, employees with high work satisfaction will actively respond to consumers’ needs, which is conducive to maintaining the long-term relationship between employees and consumers. Maintaining good relationships can prevent the loss of existing customers and help increase the number of customers.

Internal marketing as a strategic management is an effective strategy to improve internal consumer satisfaction and customer orientation [64]. Chiu et al. [26] suggested that implementing internal marketing was beneficial to improve the job satisfaction of employees in public sports centers. Additionally, Huang & Chen [27] reported that internal marketing was associated with the customer orientation of full- and part-time service employees in public sports centers. Therefore, establishing internal marketing management-centered managing means can be considered as an effective measure to make private-owned sports centers gain a stronger competitive advantage. In this study, we observed that internal communication, administrative support, and educational training were important factors that directly affect internal consumer satisfaction and indirectly affect customer orientation.

Optimizing internal communication is an effective way to improve the satisfaction of internal consumer [84]. As reported by Kim & Chung [9], effective internal communication had a positive effect on the satisfaction of the staff in the wedding hall. A study conducted by Paek [10] investigated the effects of internal marketing subordinate factors of employees in small and medium-sized enterprises on internal consumer satisfaction, and they suggested that effective internal communication was an important way to improve internal consumer satisfaction. Therefore, effective communication within an enterprise is a necessary condition for developing internal consumer satisfaction. This is to be expected, as communication is crucial to the service industry. Communication is not only between managers and employees, but also between employees and customers. Therefore, effective communication is beneficial to establish a connection between the enterprise and the customer. Additionally, we also observed an indirect effect of internal communication on customer orientation. Internal communication is essential to cultivate customer orientation of employees from customer center [84], state-owned enterprise [81], administrative organs [89], and public research institutions [68]. Therefore, effective communication within an enterprise is a necessary condition for developing internal consumer satisfaction and customer orientation.

Senior management is crucial to the development of enterprises. The manager’s encouragement and praise to employees helps to improve their enthusiasm for work. As observed in this study, administrative support is not only the direct reason to improve internal consumer satisfaction, but also the indirect reason to encourage customer orientation. Choi & Shim [41] demonstrated that administrative support had positive effects on customer orientation of civil servants in local autonomous organizations. Employees with high customer orientation are an asset. Effective personal promotion practice is the key to keeping these employees [66]. Therefore, designing and implementing administrative support is considered as an effective strategy to improve internal consumer satisfaction and customer orientation.

For the service industry, educational training can provide professional skills for employees and enhance their sensitivity to customer needs. Studies reported that internal consumers’ satisfaction with wedding hall staff has been improved through educational training [9]. Chung et al. [75] targeted the employees engaged in medical devices to analyze the effectiveness of internal marketing, they suggested that educational training had positive effects on internal consumer satisfaction. Additionally, educational training would have positive effects on customer orientation of customer center employees [84]. In the study of Lee [67], they suggested that educational training was necessary to improve the customer orientation of teenage instructors. Nam et al. [76] conducted a study aimed at employees in engineering enterprises, they suggested that educational training had positive effects on customer orientation. Therefore, according to the results observed in the present study, we considered that educational training plays an important role in developing internal consumer satisfaction and customer orientation, so it should be paid more attention. As sport center belongs to the service industry, educational training can help employees understand each other’s role relative to others and various functions in the enterprise, thus forming an overall view of service strategy and helping employees provide services to meet customers’ needs.

## Conclusion

As the results of this study indicate, internal communication, administrative support, and educational training were important factors that affect internal consumer satisfaction and customer orientation. Additionally, customer orientation was positively affected by internal consumer satisfaction. We concluded that the implementation of internal marketing for private-owned sports centers had positive effects on internal consumer satisfaction and customer orientation. Also, we suggested that higher levels of internal consumer satisfaction would encourage employees to have higher degrees of customer orientation. Therefore, the implementation of internal marketing strategy was beneficial to the development of private-owned sports centers.

### Limitations and suggestions for future research

However, this study also had some limitations.

Firstly, the participants used in this study were only distributed in a single area (Wuhan, Hubei, China), and can’t represent all the employees of the private-owned sports center in China. Therefore, generalizing the results to the entire employees in private-owned sports centers in China would be difficult.

Secondly, our investigation was focused on internal consumer satisfaction and customer orientation, not customer satisfaction and their willingness to spend. It may be more beneficial to improve the commercial value of the investigation by conducting more surveys on customer behavior.

Thirdly, the method used in this study was only quantitative questionnaire survey. Qualitative survey of internal consumers’ opinions was beneficial to formulate targeted strategies.

To the best of our knowledge, this was the first study to explore how internal consumer satisfaction and customer orientation were affected by internal marketing strategy in a Chinese private-owned sports center. This study provided evidence for formulating the operation strategy of a private-owned sports center focused on internal consumers. Future surveys should conduct in-depth interviews and focus group surveys on internal consumer, which would help managers to better understand the thoughts of internal consumers. Therefore, our study should be considered to be exploratory.

### Contribution of the research

The results obtained in this study supported the perspective that managers should regard employees as internal consumers. For the service industry, managers should pay more attention to optimizing internal communication, administrative support, and educational training, as these factors are important for improving internal consumer satisfaction and encourage customer orientation. Additionally, managers should regard internal consumer satisfaction as a key factor, as a higher level of internal consumer satisfaction will encourage employees to have higher degrees of customer orientation. To establish a competitive private-owned sports center, implementing internal marketing strategies is needed.

## Supporting information

S1 Table Compositions of questionnaire. (DOCX)

S2 Table Factors and reliability analysis of internal marketing. (DOCX)

S3 Table Factors and reliability analysis of internal consumer satisfaction. (DOCX)

S4 Table Factors and reliability analysis of customer orientation. (DOCX)
