# Supplementary material for: Internal marketing analysis for improving the internal consumer satisfaction and customer orientation of employees in private-owned sports center
Source: PLoS One. 2023 Aug 10;18(8):e0286021. doi: 10.1371/journal.pone.0286021 (PMC10414590; doi:10.1371/journal.pone.0286021)
Supplement: S1 Table — (DOCX) [file pone.0286021.s001.docx]

| **Supplementary table 1.** Compositions of questionnaire | |
| --- | --- |
| Variables | Questions |
| Sociodemographic variables, questions, and scales used in the questionnaires | |
| Age (years) | How old are you? |
| Gender (1: male; 2: female) | What is your gender? |
| Work experience (1: less than 1 year; 2: 1-2 year; 3: 3-4 year; 4: more than 4 years) | How long is your working experience? |
| Employee type (1: formal employee; 2: informal employee) | What is your employment type in the sports center? |
| Variables, questions, and scales of internal marketing used in the questionnaires (Lee, 2006; Sun, 2021) | |
| Internal communication (1-5: definitely not – it will) | Do you think our company can freely express their wishes in a free atmosphere? |
|  | Do you think that employees in our company can communicate freely about work and things other than work? |
|  | Do you think our company provides all kinds of work-related guidelines and information on work performance results? |
|  | Do you think I am free to report to my boss and express my intention actively? |
| Administrative support (1-5: definitely not – it will) | Do you think the management has created an approachable atmosphere? |
|  | Do you think management encourages open communication? |
|  | Do you think management involves employees in the planning and decision-making process? |
|  | Do you think the management has made efforts to promote the exchange of information between each other throughout the process? |
| Encouraging system (1-5: definitely not – it will) | Do you think our company uses the results of customer evaluation to reward employees? |
|  | Do you think our company rewards employees for building close customer relationships? |
|  | Do you think incentives offer employees who help improve job creativity? |
| Educational training (1-5: definitely not – it will) | Do you think service and job-related education and training are more frequent each year? |
|  | Do you think the company offers opportunities for ongoing education and training? |
|  | Do you think education and training will help me understand customers' present and future requirements? |
|  | Do you think the company's formal education and training are valuable? |
| Authority appointment (1-5: definitely not – it will) | Do you think our company allows employees to have autonomy in job-related decisions? |
|  | Do you think our company encourages employees to use their own judgment in solving problems? |
|  | Do you think our company encourages innovation? |
|  | Do you think our company gives employees a lot of initiative? |
| Variables, questions, and scales of Internal consumer satisfaction used in the questionnaires (Lee, 2007; Kim, 2016) | |
| Internal consumer satisfaction (1-5: definitely not – it will) | Do you feel about the quality of the company's internal service? |
|  | Are you satisfied with your job at the company? |
|  | Do you think your job is up to your level of competence? |
|  | Do you think your workload is just right? |
|  | Do you think employees are free to express their opinions about their work? |
|  | Do you think employees can communicate freely with their superiors about their work? |
|  | Are you satisfied with the level of treatment offered by the company? |
|  | Are you satisfied with your colleagues? |
|  | Are you satisfied with your current working environment? |
|  | Are you generally satisfied with your work in the company? |
| Variables, questions, and scales of Customer orientation used in the questionnaires (Lee, 2011; 2017) | |
| Customer orientation (1-5: definitely not – it will) | Do you put your customers first? |
|  | Will you explain it to the customer in detail? |
|  | Do you take customers' problems seriously? |
|  | Do you know what customers need in advance? |
|  | Do you understand the needs of your customers? |
|  | Are you good at listening to customers? |
|  | Are you more interested in customer behavior? |
|  | Do you recognize and respond positively to customer needs in advance? |
|  | Do you serve customers accurately? |
|  | Can you provide a good service that customers can trust? |
|  | Will you provide service at the appointed time with the customer? |
|  | Do you give individual attention to your customers? |
|  | Do you remember the customer's name? |
|  | Do you try to maintain a good relationship with your customers? |
