# Supplementary material for: Internal marketing analysis for improving the internal consumer satisfaction and customer orientation of employees in private-owned sports center
Source: PLoS One. 2023 Aug 10;18(8):e0286021. doi: 10.1371/journal.pone.0286021 (PMC10414590; doi:10.1371/journal.pone.0286021)
Supplement: S2 Table — (DOCX) [file pone.0286021.s002.docx]

| **Supplementary table 2.** Factors and reliability analysis of internal marketing | | | | | |
| --- | --- | --- | --- | --- | --- |
| Items | Composition | | | | |
|  | 1 | 2 | 3 | 4 | 5 |
| Administration support 1 | 0.875 | 0.200 | 0.132 | 0.072 | 0.133 |
| Administration support 2 | 0.860 | 0.200 | 0.205 | 0.125 | 0.150 |
| Administration support 3 | 0.857 | 0.135 | 0.214 | 0.004 | 0.086 |
| Administration support 4 | 0.851 | 0.162 | 0.206 | 0.087 | 0.095 |
| Authority appointment 2 | 0.144 | 0.885 | 0.166 | 0.140 | 0.077 |
| Authority appointment 1 | 0.179 | 0.868 | 0.156 | 0.154 | 0.058 |
| Authority appointment 4 | 0.211 | 0.852 | 0.220 | 0.132 | 0.102 |
| Authority appointment 3 | 0.196 | 0.781 | 0.261 | 0.200 | 0.144 |
| Educational training 1 | 0.148 | 0.221 | 0.858 | 0.127 | 0.198 |
| Educational training 2 | 0.212 | 0.207 | 0.853 | 0.133 | 0.188 |
| Educational training 3 | 0.237 | 0.187 | 0.835 | 0.156 | 0.172 |
| Educational training 4 | 0.287 | 0.249 | 0.777 | 0.219 | 0.148 |
| Internal communication 2 | 0.058 | 0.183 | 0.066 | 0.866 | 0.277 |
| Internal communication 4 | 0.073 | 0.084 | 0.176 | 0.835 | 0.151 |
| Internal communication 3 | 0.122 | 0.132 | 0.131 | 0.814 | 0.222 |
| Internal communication 1 | 0.029 | 0.182 | 0.157 | 0.809 | 0.142 |
| Encouraging system 2 | 0.136 | 0.100 | 0.210 | 0.243 | 0.855 |
| Encouraging system 1 | 0.157 | 0.098 | 0.116 | 0.287 | 0.826 |
| Encouraging system 3 | 0.139 | 0.127 | 0.294 | 0.215 | 0.797 |
| CR | 0.920 | 0.911 | 0.899 | 0.899 | 0.866 |
| AVE | 0.741 | 0.718 | 0.691 | 0.691 | 0.683 |
| Intrinsic value | 3.388 | 3.314 | 3.303 | 3.188 | 2.416 |
| Dispersion | 17.832 | 35.273 | 52.657 | 69.436 | 82.149 |
| Kaiser-Meyer-Olkin = 0.908; Bartlett X^2^ = 5196.675; df = 171, *P* < .001 | | | | | |
| Cronbach’s α | 0.929 | 0.929 | 0.938 | 0.900 | 0.890 |
