# Supplementary material for: Internal marketing analysis for improving the internal consumer satisfaction and customer orientation of employees in private-owned sports center
Source: PLoS One. 2023 Aug 10;18(8):e0286021. doi: 10.1371/journal.pone.0286021 (PMC10414590; doi:10.1371/journal.pone.0286021)
Supplement: S3 Table — (DOCX) [file pone.0286021.s003.docx]

| **Supplementary table 3.** Factors and reliability analysis of internal consumer satisfaction | |
| --- | --- |
| Items | Composition |
| Internal consumer satisfaction 7 | 0.887 |
| Internal consumer satisfaction 2 | 0.874 |
| Internal consumer satisfaction 6 | 0.874 |
| Internal consumer satisfaction 9 | 0.865 |
| Internal consumer satisfaction 10 | 0.862 |
| Internal consumer satisfaction 8 | 0.859 |
| Internal consumer satisfaction 3 | 0.859 |
| Internal consumer satisfaction 5 | 0.857 |
| Internal consumer satisfaction 1 | 0.846 |
| Internal consumer satisfaction 4 | 0.842 |
| CR | 0.967 |
| AVE | 0.744 |
| Intrinsic value | 7.439 |
| Dispersion | 74.392 |
| Kaiser-Meyer-Olkin = 0.949; Bartlett X^2^ = 3217.665; df = 45, *P* < .001 | |
| Cronbach’s α | 0.962 |
