# Supplementary material for: Internal marketing analysis for improving the internal consumer satisfaction and customer orientation of employees in private-owned sports center
Source: PLoS One. 2023 Aug 10;18(8):e0286021. doi: 10.1371/journal.pone.0286021 (PMC10414590; doi:10.1371/journal.pone.0286021)
Supplement: S4 Table — (DOCX) [file pone.0286021.s004.docx]

| **Supplementary table 4.** Factors and reliability analysis of customer orientation | |
| --- | --- |
| Items | Composition |
| Customer orientation 6 | 0.920 |
| Customer orientation 10 | 0.916 |
| Customer orientation 3 | 0.908 |
| Customer orientation 2 | 0.906 |
| Customer orientation 9 | 0.902 |
| Customer orientation 4 | 0.900 |
| Customer orientation 8 | 0.886 |
| Customer orientation 11 | 0.873 |
| Customer orientation 5 | 0.868 |
| Customer orientation 7 | 0.866 |
| Customer orientation 14 | 0.865 |
| Customer orientation 13 | 0.842 |
| Customer orientation 1 | 0.841 |
| Customer orientation 12 | 0.830 |
| CR | 0.980 |
| AVE | 0.776 |
| Intrinsic value | 10.860 |
| Dispersion | 77.572 |
| Kaiser-Meyer-Olkin = 0.968; Bartlett X^2^ = 5624.039; df = 91, *P* < .001 | |
| Cronbach’s α | 0.977 |
